# Potassium channel‐mediated NO‐induced vasodilation during maturation: Dominance of Kv7 channels

**DOI:** 10.1096/fba.2024-00178

**Published:** 2025-01-24

**Authors:** Anastasia A. Shvetsova, Dina K. Gaynullina, Peter Winkler, Paulus Wohlfart, Rudolf Schubert

**Affiliations:** ^1^ Faculty of Biology M.V. Lomonosov Moscow State University Moscow Russia; ^2^ Physiology, Institute of Theoretical Medicine, Faculty of Medicine University of Augsburg Augsburg Germany; ^3^ European Center of Angioscience (ECAS), Research Division Cardiovascular Physiology, Medical Faculty Mannheim Heidelberg University Mannheim Germany; ^4^ MSAT Frankfurt Sanofi Aventis Deutschland GmbH Frankfurt Germany

**Keywords:** membrane potential, arterial smooth muscle, Kv7 channel, nitric oxide, ontogenesis, vasodilation

## Abstract

Maturation represents a process characterized by adaptive changes, particularly in the circulatory system. However, it is not known whether, in neonates, potassium channels contribute to NO‐induced vasorelaxation at all and, if so, which potassium channels these are. Therefore, this study tested the hypothesis that potassium channels mediate NO‐induced vasorelaxation in newborn rats. Young (10‐ to 15‐day‐old) and adult (2‐ to 3‐month‐old) male rats were studied using real‐time PCR, isometric myography, and the sharp microelectrode technique on saphenous arteries. We observed prominent mRNA expression of several distinct isoforms of potassium channel families known to potentially mediate SNP‐induced vasodilation. Further, in both adult and young rats, SNP can relax vessels independently of potassium channels. A solely potassium channel‐independent anticontractile effect of SNP was observed also when either Kir6, or Kir2, or Kv2 channels, respectively, were available in both adult and young rats. However, when Kv1 channels were available, a Kv1 channel‐dependent component contributed to the anticontractile effect of SNP in young rats. When BK_Ca_ channels were available, a BK_Ca_ channel‐dependent component contributed to the anticontractile effect of SNP in adult rats. A considerable Kv7 channel‐dependent component contributed to the anticontractile effect of SNP in both adult and young rats. Thus, the data of the present study show for the first time that potassium channels, even multiple ones, contribute to SNP‐induced vasorelaxation in newborn rats and that the potassium channels involved in SNP‐induced vasorelaxation change from Kv1/Kv7 channels to BK_Ca_/Kv7 channels during postnatal development.

## Graphical abstract

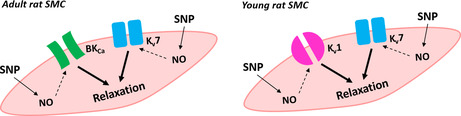

## 1 INTRODUCTION

Maturation represents a process characterized by adaptive changes in all parts of the organism, including the circulatory system. Notably, blood pressure rises about 2 times from birth to adulthood.^1^, ^2^ This adaptation of blood pressure during early postnatal development is based on numerous processes of structural and functional remodeling of the circulatory system.^3^, ^4^, ^5^ For example: (i) the anticontractile effect of the endothelium decreases,^6^, ^7^ but may also increase^8^ depending on the vascular bed studied; (ii) sympathetic nerves reduce arterial smooth muscle Ca^2+^‐sensitivity,^9^ (iii) mRNA and miRNA expression changes in a correlated manner,^10^ (iv) Rho‐kinase‐mediated calcium sensitivity decreases,^2^, ^11^, ^12^ (v) BK_Ca_ channel contribution to vascular tone regulation increases,^13^ whereas Kv1, Kv7.4, and Kir2‐channel mediated negative feedback regulation of vasocontraction is reduced, in the case of Kv7 channel partly due to an increasing role of BK_Ca_ channels^14^, ^15^ and Kv7.1 channels contribute exclusively to renal autoregulation in neonates,^16^ and (vi) the TASK‐1 channel mediated negative feedback regulation of vasocontraction is lost.^17^ Interestingly, the latter findings suggest that the contribution of potassium channels to negative feedback regulation of vasocontraction is characterized by a switch from Kir2, Kv1, BK_Ca_, Kv7, and TASK‐1 channels to Kir2, Kv7, and BK_Ca_ channels during maturation.^15^ Notably, maturational changes in the mechanisms of vasorelaxation have so far been studied in much less detail.

In addition to vasocontraction, vasorelaxation is another important process for vascular tone regulation. Vasorelaxation in smooth muscle is mediated by two major pathways, the cAMP/PKA and the cGMP/PKG pathways. Of note, the vascular smooth muscle potassium channels mentioned above are important targets of cAMP/PKA and cGMP/PKG signaling.^18^ These pathways are activated by numerous vasoactive substances. Interestingly, previous studies propose that one of these vasoactive substances, nitric oxide (NO), may be of special interest. NO is one of the major factors permanently released by endothelial cells. It exerts an enhanced anticontractile effect after birth, which presumably contributes to the mechanisms that enable the protectively low blood pressure at this age.^6^ In addition, the contribution of NO to endothelium‐dependent relaxation is affected by maturation and either increases^19^ or decreases^3^, ^6^, ^20^ depending on the vascular bed. Moreover, in adults it has been shown that NO‐induced dilation can be mediated by different potassium channels like voltage‐gated potassium channels,^21^ in particular Kv7 channels^22^ (but not in all vessels or species studied^23^); BK_Ca_ channels^24^, ^25^ in many vascular beds (for a comprehensive overview and evidence for vessels and/or species with BK_Ca_ channels resistant to NO‐induced regulation see e.g.,^18^), Kir2 channels,^26^ and Kir6 channels^27^ (for further examples as well as evidence for vessels and/or species with Kir6 channels resistant to NO‐induced regulation see e.g.,^18^). However, it is not known whether, in neonates, potassium channels contribute to NO‐induced vasorelaxation at all and, if so, which potassium channels these are. Therefore, this study tested the hypothesis that potassium channels mediate NO‐induced vasorelaxation in newborn rats.

## 2 MATERIALS AND METHODS

### 2.1 Animals

In this study male Wistar rats of two different ages were utilized: adult (2‐ to 3‐month‐old, weight 250–350 g) and young (10‐ to 15‐day‐old, weight 20–30 g). Animals were obtained from Janvier (France) or the Institute of General Pathology and Pathophysiology (Russia). Rats were provided with food and water ad libitum and were housed in a room with a controlled temperature and a 12/12 h light/dark cycle. At the day of the experiment rats were anesthetized by CO_2_ (for adult animals) and killed by decapitation. Animal studies are reported in compliance with the ARRIVE guidelines 2.0.^28^, ^29^ All experimental procedures used in this study were approved by German and Russian institutional committees on animal welfare (I‐17/17 and 93‐g, respectively). Rats have been used for research on potassium channel function in both adult and early postnatal ontogenesis in many studies (for example see^15^, ^30^). Since previous studies on this topic used males only, we also conducted experiments on males in this first study on vasorelaxation mechanisms in maturation. To identify sex differences (if any), further detailed studies need to be conducted in females, which is beyond the topic of the present study.

### 2.2 Wire myograph experiments

Saphenous arteries were carefully cleaned from surrounding tissue, cut into 2‐mm‐long segments and mounted in a wire myograph (620 M, DMT A/S, Denmark) in the preparation solution containing (mM): NaCl 145; KCl 4.5; CaCl_2_ 0.1; MgSO_4_ 1.0; NaH_2_PO_4_ 1.2; EDTA 0.025; HEPES 5.0 (pH = 7.4). The endothelium was gently removed using a rat whisker. Thereafter the solution in the myograph chambers was replaced by experimental solution containing (mM): NaCl 120; NaHСO_3_ 26; KCl 4.5; CaCl_2_ 1.6; MgSO_4_ 1.0; NaH_2_PO_4_ 1.2; D‐glucose 5.5; EDTA 0.025; HEPES 5.0 (pH = 7.4). The chambers were heated up to 37°C and continuously aerated with a mix of 5% CO_2_ + 95% O_2_ to maintain pH at 7.4 during the experiment. Data were recorded at 10 Hz using an analogue‐to‐digital converter (E14‐140М, L‐CARD, Russia) and the PowerGraph3.3 software (DISoft, Russia) or the PowerLab 4/30 system (ADInstruments, USA) and the LabChart software (ADInstruments, USA). Each arterial segment was stretched to 0.9d_100_ (90% of the inner diameter it would have at a transmural pressure of 100 mmHg), corresponding to maximum active force development.^31^

At the beginning of each experiment a standard activation procedure was performed: (1) methoxamine (α_1_‐adrenoceptor agonist, 10 μM) during 5 min following by acetylcholine (10 μM, the absence of a dilatory response confirmed successful endothelium denudation); (2) high potassium solution containing (mM): NaCl 6; NaHСO_3_ 26; KCl 118.5; CaCl_2_ 1.6; MgSO_4_ 1.0; NaH_2_PO_4_ 1.2; D‐glucose 5.5; EDTA 0.025; HEPES 5.0 (pH = 7.4) during 5 min; (3) methoxamine (10 μM) during 5 min.

The experimental protocol consisted of two concentration‐response relationships to methoxamine (concentration range from 0.01 μM to 30 μM for 10‐ to 15‐day‐old rats or from 0.01 μM to 10 μM for adult rats, duration at each concentration was 3 min). After the end of the activation procedure, two arterial preparations were treated with a combination of potassium channel blockers, another two preparations with a 50 mM potassium solution containing (mM): NaCl 74.5; NaHСO_3_ 26; KCl 50; CaCl_2_ 1.6; MgSO_4_ 1.0; NaH_2_PO_4_ 1.2; D‐glucose 5.5; EDTA 0.025; HEPES 5.0 (pH = 7.4) for 20 min. Thereafter, the first concentration–response relationship to methoxamine was obtained.

Subsequently, sodium nitroprusside (SNP) 0.1 μMol L^−1^ was added for 10 min to one arterial segment previously treated with the mixture of potassium channel blockers and to another arterial segment previously treated with 50 mM potassium solution. Then the preparations were incubated during 20 min with the mixture of potassium channel blockers or 50 mM potassium solution (the same preparations as before during the first concentration–response relationship), followed by the second concentration–response relationship to methoxamine (shown in Figures).

In some cases, arterial preparations previously treated with the mixture of potassium channel blockers were treated further. After wash‐out, SNP 0.1 μM was added to the same chamber as before. Ten minutes later both arterial segments were treated for 20 min with the same mixture of potassium channel blockers as before. Thereafter, a single potassium channel blocker of interest (not included to the mixture used previously) was added to the chambers for 10 min.

The following potassium channel blockers were used in this study: DPO‐1 (1 μM, blocks Kv1 channels^32^, ^33^), stromatoxin (0.1 μM, blocks Kv2 channels^34^), XE991 (3 μM, blocks Kv7 channels^35^, ^36^), BaCl_2_ (30 μM, blocks Kir2 channels^26^), glibenclamide (3 μM, blocks K_ATP_ channels^37^), iberiotoxin (0.1 μM, blocks BK_Ca_ channels^38^).

**FIGURE 1:**
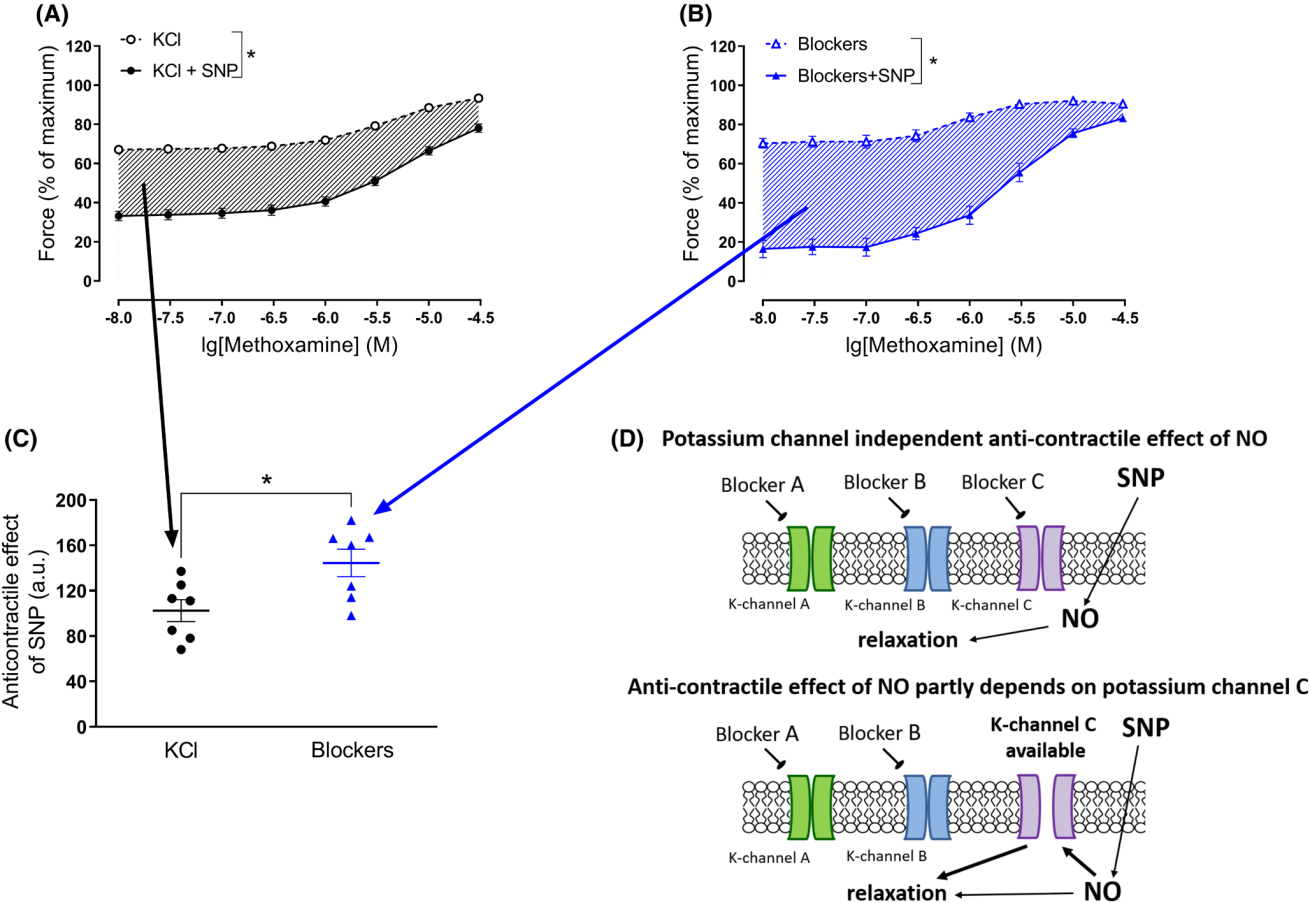
Demonstration of how the anticontractile effect of SNP was assessed. For each individual preparation treated with 50 mM potassium solution, the differences between the AUCs with and without SNP (difference shown in A as hatched area) are determined; attention, here mean data are shown for illustration purposes. The same procedure is used when preparations were treated with a mixture of potassium channel blockers with and without SNP (difference shown in B as hatched area). The individual AUCs are plotted in panel C. Panel D shows a scheme explaining the experimental protocol when vessels were treated with a potassium channel blocker mixture leaving just one potassium channel available to study its role in the anticontractile effect of SNP.

Vessel reactivity was expressed as active force. To calculate active force values at each time point of interest, the force value at the fully relaxed state was subtracted from all recorded data. All active force values were expressed as the percentage of the maximum active force developed during the respective first concentration–response relationship to methoxamine. Further, area under the curve (AUC) values were calculated for the second concentration–response relationships to methoxamine in GraphPad Prizm 9.5.1 (La Jolla, CA, USA). The difference between the AUCs for preparations treated with 50 mM potassium solution/mixture of potassium channel blockers with and without SNP were used to assess the anticontractile effect of SNP (see Figure 1 for illustration).

Along with NO, SNP has been shown to release cyanide in vascular wall.^39^ Importantly, our previous data indicate that NO, rather than cyanide, mediates the anticontractile and dilatory effects of SNP, as (i) cyanide itself has no anticontractile effect, and (ii) inactivators of cyanide do not modify the anticontractile or dilatory effects of SNP in vessels.^26^, ^40^

### 2.3 Membrane potential measurements

Simultaneous measurement of smooth muscle membrane potential and of isometric force was performed using the sharp microelectrode technique as described previously.^15^, ^41^ Briefly, endothelium‐denuded saphenous arteries were mounted in a wire myograph (model 301, DMT A/S, Denmark). At the beginning of each experiment, vessels were normalized and activated as described in the *Wire myograph experiments* section above. To exclude distortion of the electrode by gas bubbles, the experimental solution was heated to 37°C and bubbled with 5% CO_2_ in O_2_ in a separate reservoir. This solution was then supplied to the myograph chamber by a peristaltic pump (Julabo, Germany) at a rate of 2 mL/min.

For the manufacturing of the microelectrodes, aluminosilicate glass was used. They were filled with saturated KCl and had resistances of 30 to 70 MOhm. The resistance of the electrode was continuously monitored by applying subthreshold electrical pulses (20 mV amplitude, 25 ms duration) at a frequency of 1 Hz. Membrane potential recordings were accepted when the following criteria were fulfilled (i) a sharp drop of the potential on cell penetration; (ii) a stable level of the membrane potential recording for at least 30 s; (iii) a return to the zero‐potential level after electrode removal; (iv) a similar electrode resistance before and after the measurement.^41^

Membrane potential was measured under the following experimental conditions: (1) in the presence of 50 mM potassium solution (KCl); (2) in the presence of 50 mM potassium solution together with SNP 0.1 μM (KCl + SNP); (3) after washout (CON); (4) in the presence of the mixture of potassium channel blockers DPO‐1 + stromatoxin + BaCl_2_ + glibenclamide + iberiotoxin (Blockers); (5) in the presence of the same mixture of potassium channel blockers together with SNP 0.1 μM (Blockers+SNP); (6) in the presence of the same mixture of potassium channel blockers together with XE991 + SNP 0.1 μmol L^−1^ (Blockers+SNP+XE991).

Active force values were calculated as the percentage of the maximum force obtained during the concentration–response relationship to methoxamine.

### 2.4 Gene expression analysis

Saphenous arteries were isolated as described in previous sections, snap frozen in liquid nitrogen and stored at −80°C. Total RNA was isolated using the RNeasy RNA isolation kit and purified according to the manufacturer's instruction. Control of the quantity and quality of the isolated RNA was performed using an RNA 6000 nano kit (Agilent, Waldbronn, Germany). Further analysis was executed only for samples with an integrity RIN value >7.5. For reverse transcription a high‐capacity RNA‐to‐cDNA Kit was used (Applied Biosystems, Weiterstadt, Germany). The expression of potassium channel genes was determined by real‐time PCR in parallel including four reference (housekeeping) genes employing TaqMan microfluidic card technology in a Viia7 thermocycler (ThermoFisher, Darmstadt, Germany) with a maximum of 40 cycles. All TaqMan primers were tested before by dilution experiments and used only when amplification efficacies were close to 100%. Threshold quantification cycles (Cq values) were obtained for each gene by the manufacturer's Viia7 software and further analyzed using the ArrayStudio software package (Version 9, Omicsoft Corporation, Research Triangle Park, NC, United States). The level of gene expression was determined by first subtracting a geometric mean value of the four reference genes *B2M*, *Eif2b1*, *Gusb*, and *Ywhaz* from individual Cq values. Relative expression was then calculated as potency of this difference in Cq to base 2.

### 2.5 Drugs

Methoxamine, acetylcholine (all dissolved in H_2_O), XE991, and glibenclamide (dissolved in DMSO), as well as all salts were obtained from Sigma. DPO‐1 (dissolved in DMSO) was obtained from Tocris. Iberiotoxin and stromatoxin (dissolved in H_2_O) were obtained from Alomone Labs. BaCl_2_ (dissolved in H_2_O) was obtained from Riedel‐de‐Haёn.

### 2.6 Data and statistical analysis

The data and statistical analysis comply with the recommendations on experimental design and analysis in pharmacology; for details see.^42^ Treatment of arterial segments with certain substances within each experimental group was randomized. Blinding of the operator was not feasible because vessel responses observed by the operator to manage the experiment permitted inferences about the treatment. However, data analysis was performed semi‐blinded by an independent analyst.

Statistical analysis was performed using GraphPad Prism 9.5.1. The normality of the data distribution was tested using the Shapiro–Wilk test. Data are presented as mean and SEM (if data distribution was normal) or as median and the interquartile range or 95% confidence interval (if data distribution was different from normal); *n* represents the number of animals, that is, biological replicates.

Concentration–response relationships to methoxamine between two groups were compared using repeated measures ANOVA. Statistical analyses of SNP's anticontractile effect and of the effect of a particular blocker on vessel tone were performed using a two‐sided unpaired Student's *t*‐test or Mann–Whitney *U*‐test, depending on the type of data distribution. Statistical analyses of membrane potential values and corresponding values of tone were performed using a paired Student's *t*‐test. Differences were accepted as statistically significant if the *p*‐value was less than 0.05.

## 3 RESULTS

### 3.1 mRNA expression of isoforms of potassium channels in rat saphenous arteries

**FIGURE 2:**
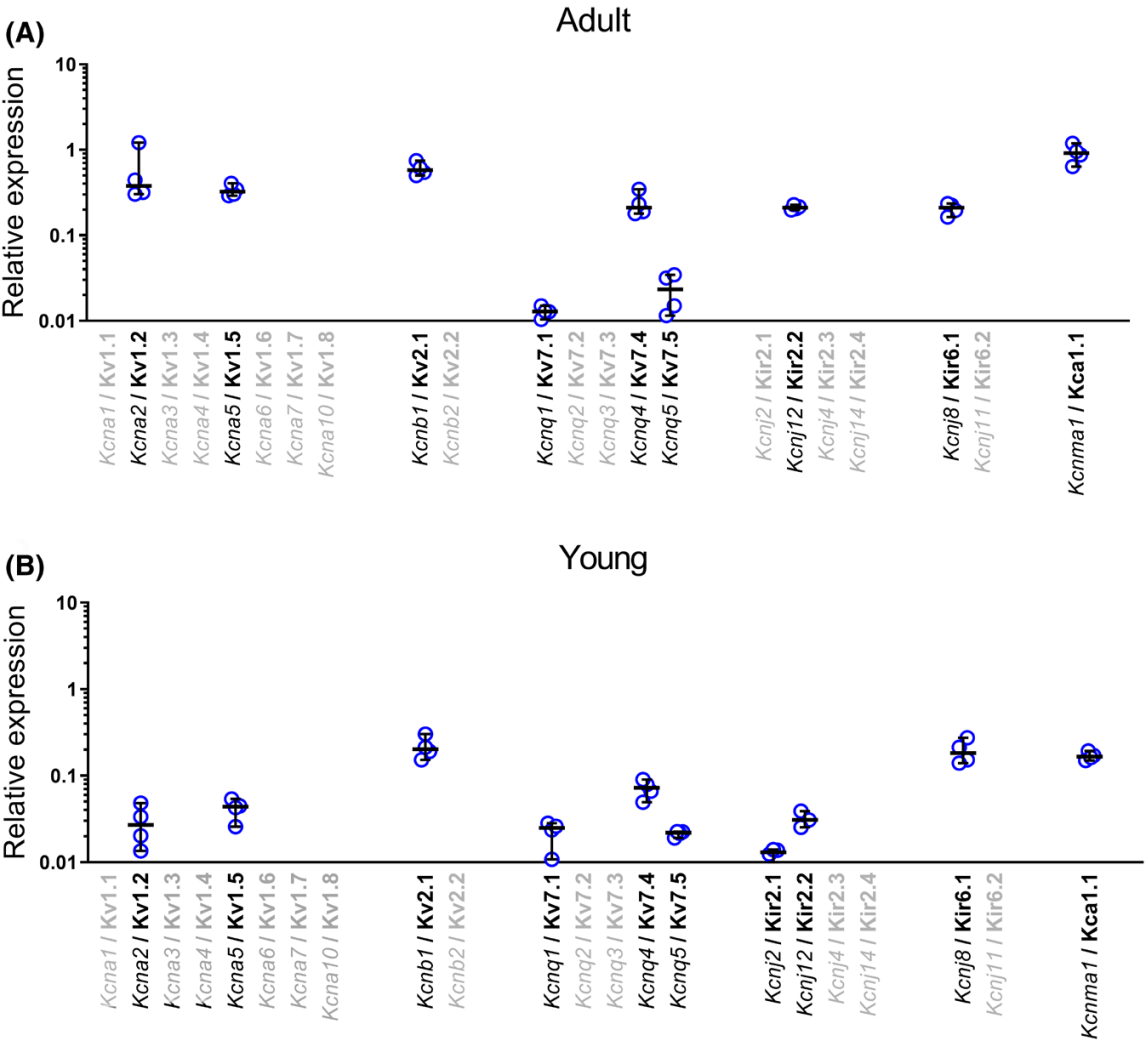
mRNA expression of isoforms of potassium channels, known to potentially mediate SNP‐induced vasodilation, in saphenous arteries from adult (A) and young (B) rats. *Kcna1‐7*, Kv1.1‐Kv1.7; *Kcna10*, Kv1.8; *Kcnb1‐2*, Kv2.1‐Kv2.2; *Kcnq1‐5*, Kv7.1‐Kv7.5; *Kcnj2*, Kir2.1; *Kcnj12*, Kir2.2; *Kcnj4*, Kir2.3; *Kcnj*14, Kir2.4; *Kcnj8*, Kir6.1; *Kcnj11*, Kir6.2; *Kcnma1*, KCa1.1. Relative expression is reported in relation to the expression of four reference (housekeeping) genes, *B2M*, *Eif2b1*, *Gusb*, and *Ywhaz.* . Data are presented as the median with 95% confidence interval, n=4.

The analysis of mRNA expression focused on potassium channels, which are known to potentially mediate SNP‐induced vasodilation, namely on Kv1 (*Kcna*), Kv2 (*Kcnb*), Kv7 (*Kcnq*), Kir2 (*Kcnj* (2, 12, 4, 14)), Kir6 (*Kcnj* (8, 11)), and BK_Ca_ (*Kcnma1*) channels. In saphenous arteries of both adult and young rats, the expression of at least a few distinct isoforms was found for each of these potassium channels (Figure 2).

### 3.2 Effect of the NO‐donor SNP on rat saphenous arteries

**FIGURE 3:**
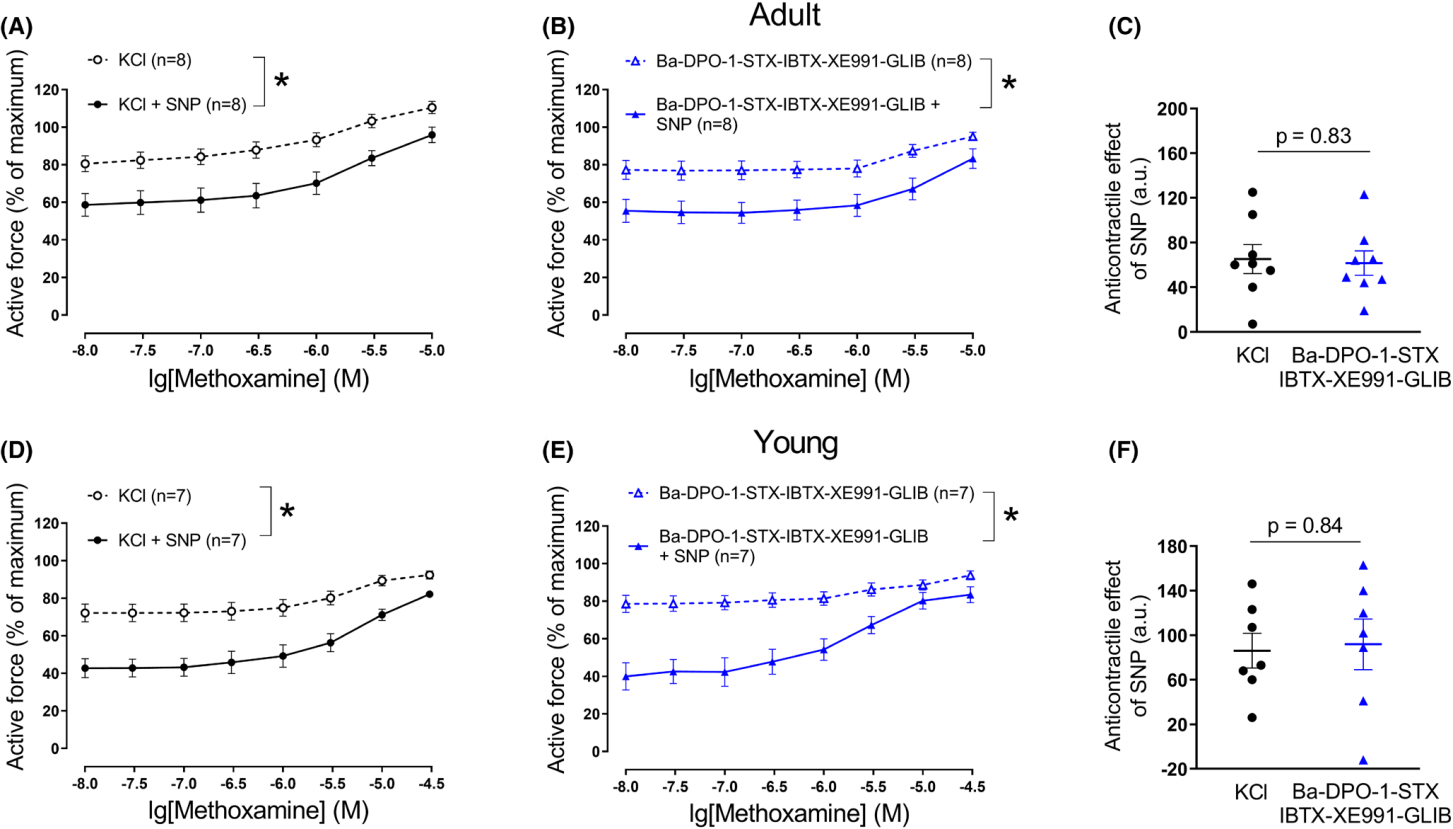
Anticontractile effect of SNP under conditions of potassium channel blockade. Concentration–response relationships for methoxamine in the presence of 50 mM KCl alone or of KCl and SNP (0.1 μM) from arteries of adult (A) and young rats (D), respectively. *p < 0.05 (repeated measures ANOVA). Concentration–response relationships for methoxamine in the presence of a combination of potassium channels blockers (BaCl_2_ (Ba) 30 μM, DPO‐1 1 μM, stromatoxin (STX) 0.1 μM, iberiotoxin (IBTX) 0.1 μM, XE991 3 μM, glibenclamide (GLIB) 3 μM) without and with SNP (0.1 μM) from arteries of adult (B) and young rats (E), respectively. *p < 0.05 (repeated measures ANOVA). Anticontractile effect of SNP, presented as the difference between the area under the curves in the presence of KCl alone and together with SNP (circles) and in the presence of potassium channels blockers alone and together with SNP (triangles) from arteries of adult (C) (unpaired Student's *t*‐test) and young rats (F) (unpaired Student's *t*‐test).

Treatment of arterial segments with 50 mM KCl increases the extracellular concentration of potassium ions, so that the electrochemical gradient for potassium is greatly reduced. Under these conditions, vessel reactivity is largely independent of potassium channels even when potassium channels are open. Addition of 50 mM KCl was associated with an increase in active force of adult saphenous arteries and the additional administration of ascending concentrations of the α_1_‐adrenoceptor agonist methoxamine caused a further vasocontraction (Figure 3A). The NO‐donor SNP (0.1 μM) weakened this contraction (Figure 3A).

In arteries of adult rats, administration of potassium channel blockers (DPO‐1, stromatoxin, XE991, BaCl_2_, glibenclamide, and iberiotoxin) and the additional administration of increasing concentrations of methoxamine produced a similar level of vessel tone as observed using 50 mM potassium chloride and methoxamine (Figure 3B) (*n* = 8; *p* = 0.08, repeated measures ANOVA). SNP also weakened this contraction (Figure 3B). We did not detect any relevant difference in the effect of SNP between vessels treated with 50 mM potassium chloride and methoxamine or treated with the mixture of potassium channel blockers and methoxamine (Figure 3C). Importantly, qualitatively and quantitatively similar results were observed in experiments on saphenous arteries of young rats (Figure 3D–F).

These data indicate that, at least with respect to the anticontractile effect of SNP, the blocker mixture contained all the necessary blockers to mimic the effect of general functional removal of potassium channel‐mediated effects by 50 mM potassium solution. These data also show that at least part of the anticontractile effect of NO was independent of potassium channels.

In order to identify potassium channel‐dependent effects of SNP, we excluded selected potassium channel blocker(s) from the blocker mixture in each of the subsequent series of experiments.

### 3.3 Contribution of Kir2 and Kir6 channels to the anticontractile effect of the NO‐donor SNP

**FIGURE 4:**
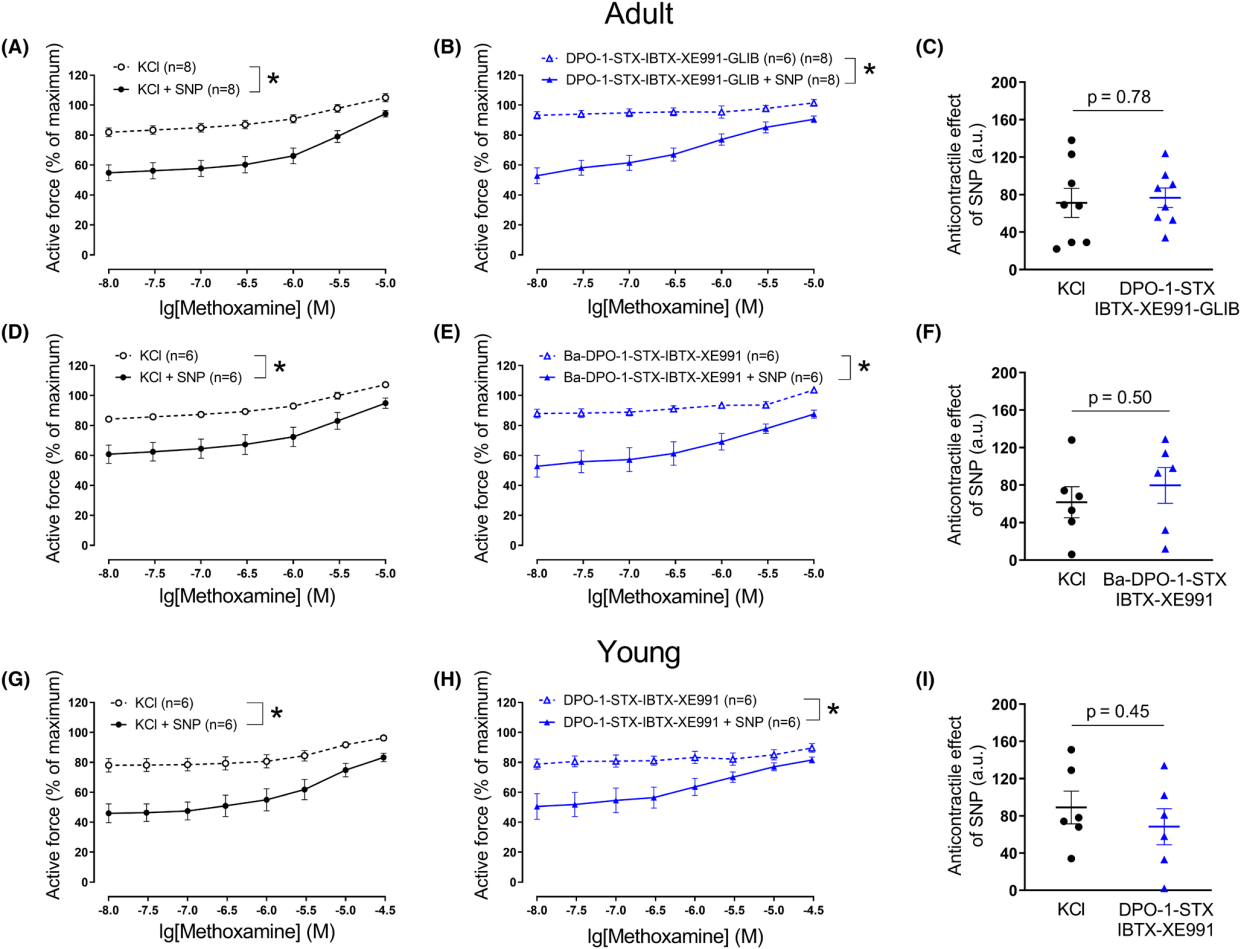
Anticontractile effect of SNP under conditions of potassium channel blockade, except Kir2 or/and Kir6 channels. Concentration–response relationships for methoxamine in the presence of 50 mM KCl alone or of KCl and SNP (0.1 μM) from arteries for adult (A, D) and young rats (G), respectively. *p < 0.05 (repeated measures ANOVA). Concentration–response relationships for methoxamine in the presence of a combination of potassium channels blockers, except BaCl_2_ (B) or glibenclamide (E) without and with SNP (0.1 μM) from arteries of adult rats. *p < 0.05 (repeated measures ANOVA). Concentration–response relationships for methoxamine in the presence of a combination of potassium channels blockers, except BaCl_2_ and glibenclamide (H) without and with SNP (0.1 μM) from arteries of young rats. *p < 0.05 (repeated measures ANOVA). Anticontractile effect of SNP, presented as the difference between the area under the curves in the presence of KCl alone and together with SNP (circles) and in the presence of corresponding potassium channels blockers alone and together with SNP (triangles) from arteries of adult (C, F) (unpaired Student's *t*‐test) and young rats (I) (unpaired Student's *t*‐test).

In this series of experiments, the NO donor SNP decreased contractile responses of arteries from adult and young rats in the presence of 50 mM KCl and methoxamine, similarly to previous series of experiments (Figure 4A,D,G).

The administration of a potassium channel blocker mixture without barium (Figure 4B) or without glibenclamide (Figure 4E) and the additional application of increasing methoxamine concentrations contracted the arterial segments of adult rats to a similar extent as was observed during treatment with 50 mM KCl and methoxamine (*n* = 8, *p* = 0.13 and *n* = 6, *p* = 0.98, respectively, repeated measures ANOVA) (Figure 4A,D).

SNP attenuated the contraction produced by the potassium channel blocker mixture without barium or glibenclamide, respectively, and methoxamine (Figure 4B,E). We did not detect any relevant difference in the anticontractile effect of SNP between adult vessels treated with 50 mM potassium chloride and methoxamine or treated with the mixture of potassium channel blockers without barium or glibenclamide, respectively, and methoxamine (Figure 4C,F).

Since omission of both barium and glibenclamide from the blocker mixture did not alter the response to SNP compared to vessels treated with 50 mM potassium chloride in adult animals, both blockers were excluded from the blocker mixture at the same time in experiments on young rats to reduce the number of animals used. This potassium channel blocker mixture and methoxamine contracted the arterial segments; this effect was comparable to the contraction induced by 50 mM KCl and methoxamine (n = 6, *p* = 0.88, repeated measures ANOVA) (Figure 4G,H).

SNP attenuated the contraction produced by the potassium channel blocker mixture without barium and glibenclamide in young arteries (Figure 4H). However, we did not detect any relevant difference in the anticontractile effect of SNP between vessels treated with 50 mM potassium chloride and methoxamine or treated with the mixture of potassium channel blockers without barium and glibenclamide and methoxamine (Figure 4I).

### 3.4 Contribution of BK_Ca_ channels to the anticontractile effect of the NO‐donor SNP

**FIGURE 5:**
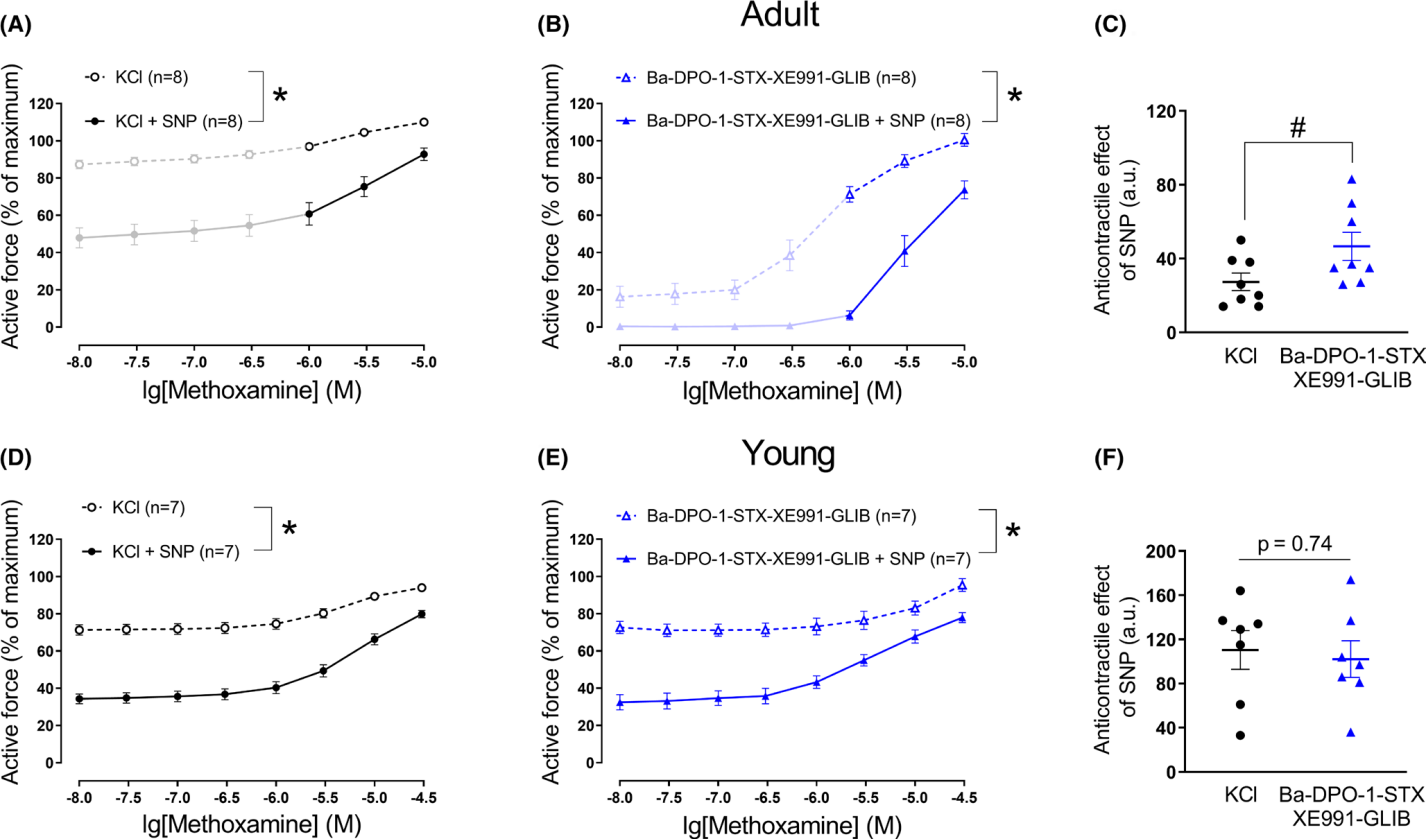
Anticontractile effect of SNP under conditions of potassium channel blockade, except BK_Ca_ channels. Concentration–response relationships for methoxamine in the presence of 50 mM KCl alone or of KCl and SNP (0.1 μM) from arteries of adult (A) and young rats (D), respectively. *p < 0.05 (repeated measures ANOVA). Concentration–response relationships for methoxamine in the presence of a combination of potassium channels blockers, except iberiotoxin, without and with SNP (0.1 μM) from arteries of adult (B) and young rats (E), respectively. *p < 0.05 (repeated measures ANOVA). Anticontractile effect of SNP, presented as the difference between the area under the curves in the presence of KCl alone and together with SNP (circles) and in the presence of corresponding potassium channels blockers alone and together with SNP (triangles) from arteries of adult (C) (unpaired Student's *t*‐test) and young rats (F) (unpaired Student's *t*‐test), #*p* < 0.05. For adult rats, only the range of methoxamine concentrations between 1 and 10 μM was used; this is shown by fading out the range of methoxamine concentrations between 0.01 and 1 μM (see results section for the justification of this approach).

Also in this series of experiments, an anticontractile effect of the NO‐donor SNP was observed in the presence of 50 mM KCl in both adult and young arteries (Figure 5A,D).

Administration of a potassium channel blocker mixture without iberiotoxin and additional application of increasing methoxamine concentrations contracted arterial segments of adult rats less than was observed using 50 mM KCl and methoxamine (*n* = 8; *p* < 0.05, repeated measures ANOVA) (Figure 5A,B).

SNP attenuated the contraction produced by the potassium channel blocker mixture without iberiotoxin in adult arteries (Figure 5B). Importantly, in a recent study, we observed a dual effect of NO‐donors on BK_Ca_ channels: they facilitate NO‐induced relaxation at higher contractility levels due to a predominant PKG‐mediated activation of BK_Ca_ channels. However, they also limit NO‐induced relaxation at intermediate and low contractility levels, which is caused by a predominant NO‐induced reduction in activator calcium influx and deactivation of the BK_Ca_ channel.^40^ Therefore, we limited the analysis of the relaxing effect of SNP in this study to higher contractility levels in the range of methoxamine concentrations between 1 and 10 μM. The anticontractile effect of SNP in the presence of the potassium channel blocker mixture without iberiotoxin and methoxamine was larger than in the presence of 50 mM of KCl and methoxamine (Figure 5C).

As in arteries of adult rats, the mixture of blockers without iberiotoxin and methoxamine increased active force in arteries of young rats. However, we did not detect any relevant difference between this effect and the effect observed in the presence of 50 mM KCl and methoxamine (*n* = 7, *p* = 0.74, repeated measures ANOVA) (Figure 5D,E). SNP attenuated the contraction produced by both the potassium channel blocker mixture without iberiotoxin and methoxamine and by 50 mM KCl and methoxamine in young arteries (Figure 5D,E). We did not detect any relevant difference in the anticontractile effect of SNP between vessels treated with 50 mM potassium chloride and methoxamine or with the mixture of potassium channel blockers without iberiotoxin and methoxamine in young rats (Figure 5F).

### 3.5 Contribution of Kv1 and Kv2 channels to the anticontractile effect of the NO‐donor SNP

**FIGURE 6:**
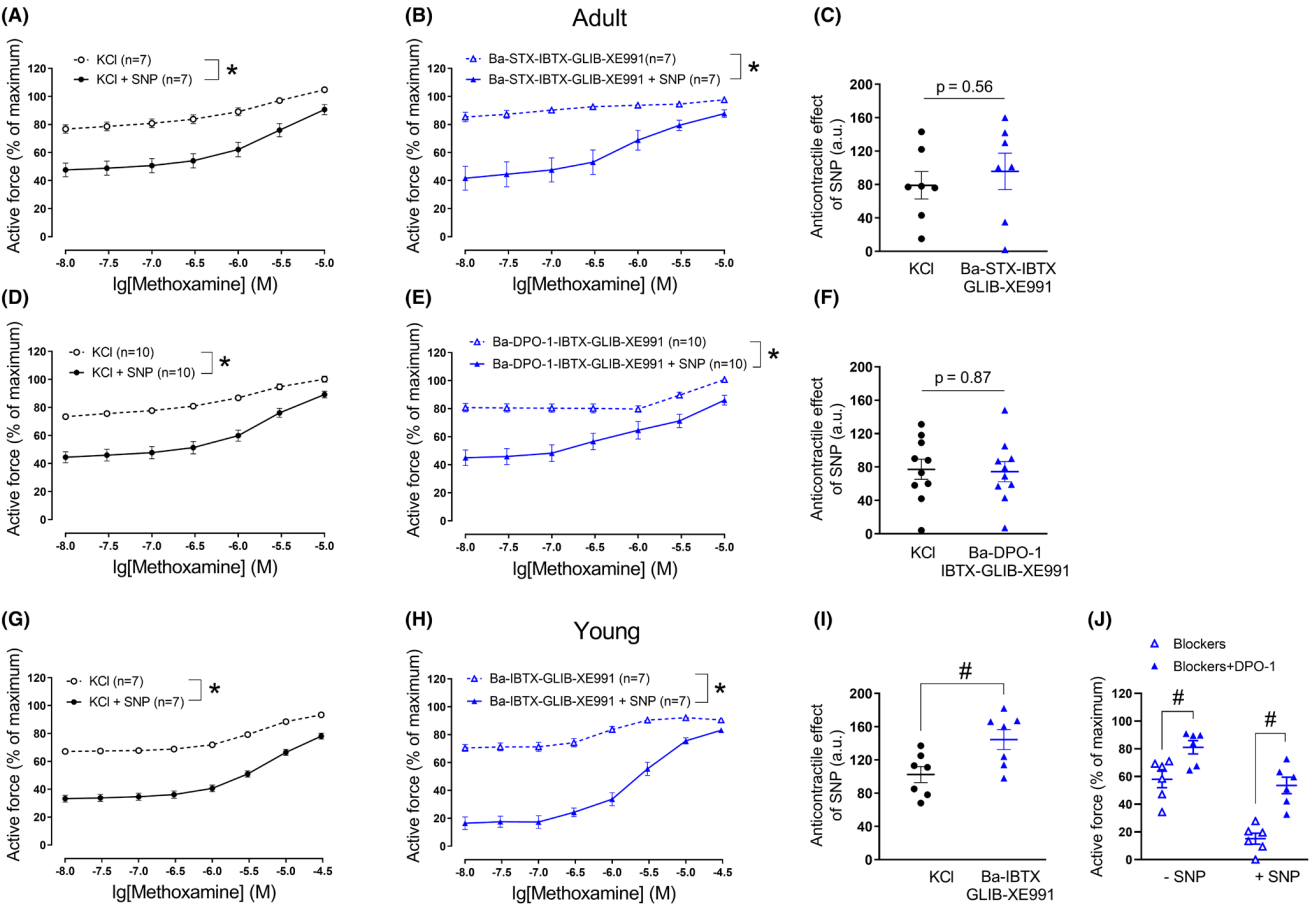
Anticontractile effect of SNP under conditions of potassium channel blockade, except Kv1 or/and Kv2 channels. Concentration–response relationships for methoxamine in the presence of 50 mM KCl alone or of KCl and SNP (0.1 μM) from arteries of adult (A, D) and young rats (G), respectively. *p < 0.05 (repeated measures ANOVA). Concentration–response relationships for methoxamine in the presence of a combination of potassium channels blockers, except DPO‐1 (B) or stromatoxin (E) without and with SNP (0.1 μM) from arteries of adult rats. *p < 0.05 (repeated measures ANOVA). Concentration–response relationships for methoxamine in the presence of a combination of potassium channels blockers, except DPO‐1 and stromatoxin (H) without and with SNP (0.1 μM) from arteries of young rats. *p < 0.05 (repeated measures ANOVA). Anticontractile effect of SNP, presented as the difference between the area under the curves in the presence of KCl alone and together with SNP (circles) and in the presence of corresponding potassium channels blockers alone and together with SNP (triangles) from arteries of adult (C, F) (unpaired Student's *t*‐test) and young rats (I) (unpaired Student's *t*‐test), #*p* < 0.05. Active force of young arteries, induced by the combination of potassium channel blockers before and after administration of 1 μM of DPO‐1 in the absence (‐SNP) and presence (+SNP) of SNP (0.1 μM, J). #*p* < 0.05 (unpaired Student's *t*‐test).

As in previous series of experiments, SNP induced an anticontractile effect when arteries were treated with 50 mM KCl and methoxamine (Figure 6A,D,G).

The administration of a potassium channel blocker mixture without DPO‐1 (Figure 6B) or stromatoxin (Figure 6E), respectively, and the additional application of increasing methoxamine concentrations contracted the arterial segments of adult rats to a similar extent as was observed during treatment with 50 mM KCl and methoxamine (*n* = 7, *p* = 0.15 and *n* = 10, *p* = 0.91, respectively, repeated measures ANOVA) (Figure 6A,D).

SNP attenuated the contraction produced by the potassium channel blocker mixture without DPO‐1 or stromatoxin, respectively, and methoxamine (Figure 6B,E). However, we did not detect any relevant difference in the anticontractile effect of SNP between vessels treated with 50 mM potassium chloride and methoxamine or treated with the mixture of potassium channel blockers without DPO‐1 or stromatoxin, respectively, and methoxamine (Figure 6C,F).

Since omission of both DPO‐1 and stromatoxin from the blocker mixture did not alter the response to SNP compared to vessels treated with 50 mM potassium chloride in adult animals, both blockers were excluded from the blocker mixture at the same time in the experiments on young rats to reduce the number of animals used. As in adult arteries, the mixture of blockers without DPO‐1 and stromatoxin and methoxamine increased active force, an effect comparable to the effects observed in the presence of 50 mM KCl and methoxamine (*n* = 7, *p* = 0.17, repeated measures ANOVA) (Figure 6G,H).

SNP attenuated the contraction produced by the potassium channel blocker mixture without DPO‐1 and stromatoxin in young arteries (Figure 6H). However, in contrast to adult arteries, the anticontractile effect of SNP in the presence of the mixture of blockers without DPO‐1 and stromatoxin and methoxamine was larger than in the presence of 50 mM of KCl and methoxamine (Figure 6I). Moreover, after precontraction of the arteries from young rats with the mixture of blockers without DPO‐1 and stromatoxin, a further increase in wall tension was detected after addition of DPO‐1 both in the absence as well as in the presence of SNP (Figure 6J). Notably, DPO‐1 contracted arteries by 22 (18–27) % in the absence of SNP and by 42 (34–44) % in the presence of SNP (*p* < 0.05, Mann–Whitney test), indicating a higher contractile effect of DPO‐1 in the presence of SNP.

### 3.6 Contribution of Kv7 channels to the anticontractile effect of the NO donor SNP

**FIGURE 7:**
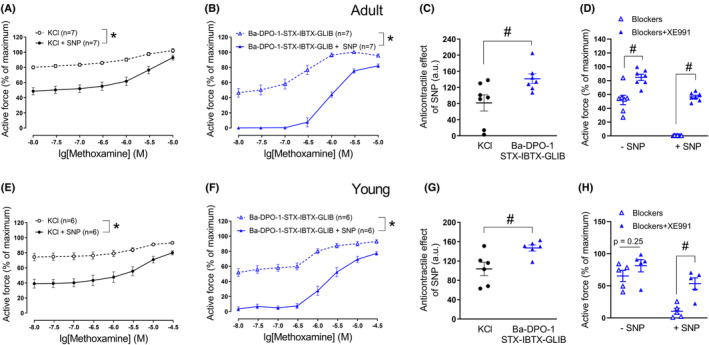
Anticontractile effect of SNP under conditions of potassium channel blockade, except Kv7 channels. Concentration–response relationships for methoxamine in the presence of 50 mM KCl alone or of KCl and SNP (0.1 μM) from arteries of adult (A) and young rats (E), respectively. *p < 0.05 (repeated measures ANOVA). Concentration–response relationships for methoxamine in the presence of a combination of potassium channels blockers, except XE991, without and with SNP (0.1 μM) from arteries of adult (B) and young (F) rats. *p < 0.05 (repeated measures ANOVA). Anticontractile effect of SNP, presented as the difference between the area under the curves in the presence of KCl alone and together with SNP (circles) and in the presence of corresponding potassium channels blockers alone and together with SNP (triangles) from arteries of adult (C) and young rats (G). #*p* < 0.05 (unpaired Student's *t*‐test). Active force of adult (D) and young (H) arteries, induced by the combination of potassium channel blockers before and after administration of 3 μM of XE991 in the absence (‐SNP) and presence (+SNP) of SNP (0.1 μM, J). #*p* < 0.05 (unpaired Student's *t*‐test).

In this series of experiments, an anticontractile effect of SNP was observed in the presence of 50 mM KCl and methoxamine both in adult and young arteries (Figure 7A,E).

Administration of a potassium channel blocker mixture without XE991 and the additional application of increasing methoxamine concentrations contracted arterial segments of adult rats less than was observed using 50 mM KCl and methoxamine (*n* = 7, *p* < 0.05, repeated measures ANOVA) (Figure 7A,B). Contractile responses to the potassium channel blocker mixture without XE991 and methoxamine in young arteries were similar compared to responses induced by 50 mM KCl and methoxamine (*n* = 6, *p* = 0.08, repeated measures ANOVA) (Figure 7E,F).

SNP attenuated the contraction produced by the potassium channel blocker mixture without XE991 in both adult and young arteries (Figure 7B,F). Importantly, this anticontractile effect of SNP in the presence of the mixture of blockers without XE991 and methoxamine was larger than in the presence of 50 mM KCl and methoxamine in both adult and young arteries (Figure 7C,G).

In addition, after precontraction of the arteries with the mixture of blockers without XE991, a further increase in wall tension was detected after addition of XE991 both in the absence as well as in the presence of SNP in both adult (Figure 7D) and young (Figure 7H) rats.

Notably, in adult arteries XE991 contracted arteries by 33 ± 4% of active force in the absence of SNP and by 56 ± 2% in the presence of SNP (*p* < 0.05 unpaired Student's *t*‐test) (Figure 7D). In young arteries, in the presence of SNP an effect of XE991 appears: XE991 сontracted arteries by 43 ± 9% (*p* < 0.05, unpaired Student's *t*‐test) (Figure 7H).

### 3.7 Contribution of Kv7 channels to the regulation of membrane potential under the influence of the NO‐donor SNP

It is well known that potassium channels are important regulators of arterial smooth muscle membrane potential. If potassium channels are involved in the anticontractile effect of SNP, corresponding changes of membrane potential must be observed. The experiments described above studying vessel contractility demonstrated that the Kv7 channel is the only channel involved in the anticontractile effect of SNP in saphenous artery of both age groups. Therefore, we focused on Kv7 channels to test the association between changes in vessel tone and membrane potential by performing additional experiments on saphenous arteries form adult and young rats with simultaneous registration of vascular tone and membrane potential.

**FIGURE 8:**
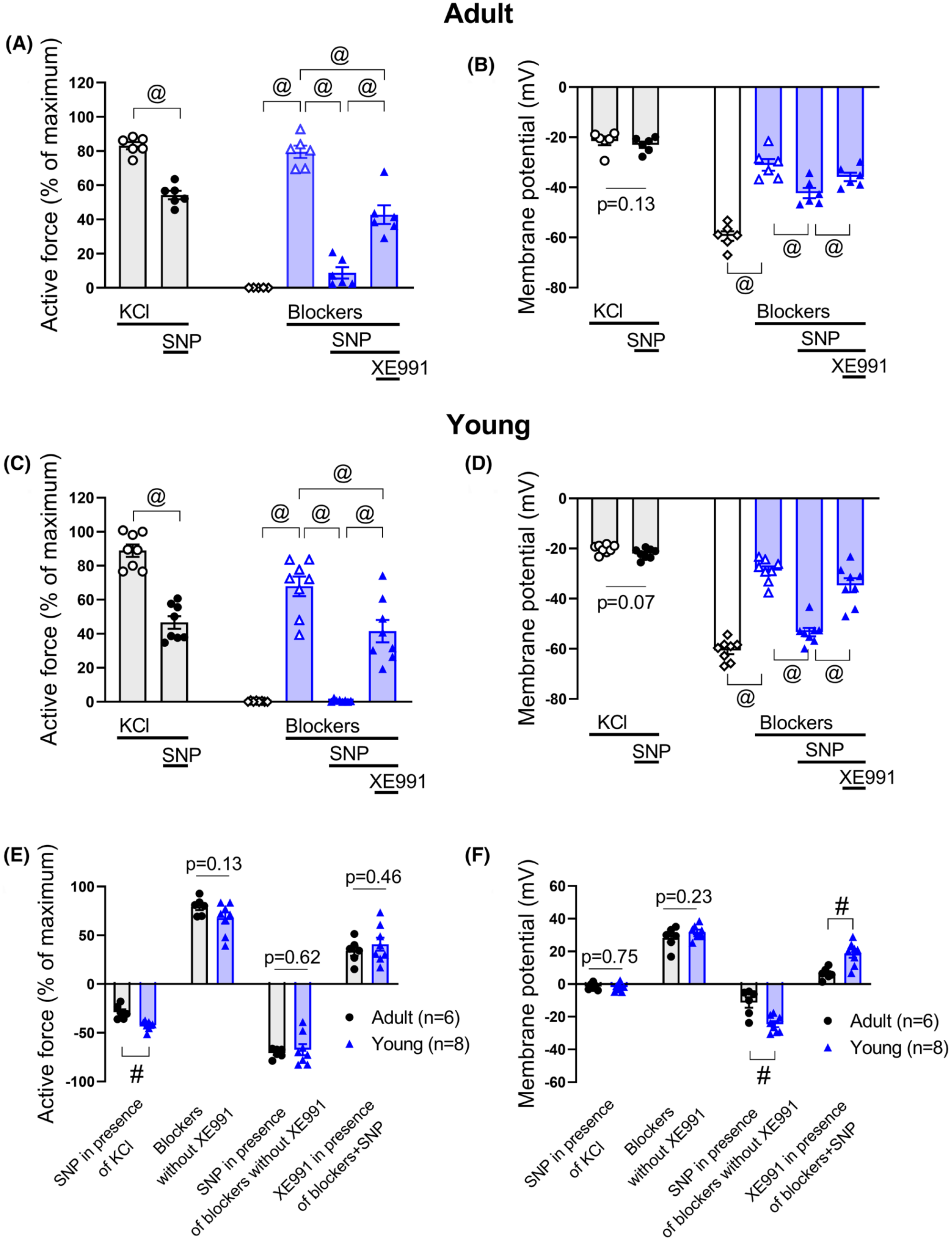
The effects of SNP on force and membrane potential in arterial smooth muscle of adult and young rats. The values of force (A, C) and membrane potential (B, D) were determined under six conditions: In 50 mM KCl alone (first bar from left in A–D) and together with SNP (0.1 μM) (second bar from left in A–D), in the absence of any substances (third bar from left in A–D), after incubation with a mixture of blockers (except XE991) without SNP (fourth bar from left in A–D), with SNP (fifth bar from left in A‐D) and after addition of 3 μM XE991 (sixth bar from left in A–D). @*p* < 0.05 (paired Student's *t*‐test). Changes in force (E) and membrane potential (F) in response to described stimuli for adult and young arteries. #*p* < 0.05 (unpaired Student's *t*‐test).

50 mM KCl caused vasocontraction and depolarization of arterial smooth muscle of both adult and young rats (Figure 8A–D). Importantly, upon further treatment with SNP we did not detect any relevant change in the membrane potential in both age groups, despite the development of vasodilation (Figure 8A–D).

In accordance with previous experimental series, the mixture of blockers without XE991 led to the development of strong vasocontraction in both adult and young arteries, which was reduced after addition of SNP (Figure 8A,C). Further treatment with XE991 contracted the arteries (Figure 8A,C). Of note, changes of vascular tone in response to these stimuli were similar in adult and young arteries (Figure 8E).

Corresponding shifts of membrane potential accompanied changes of tone. The resting membrane potential of arterial smooth muscle was −59.4 ± 1.9 mV in adult rats and −60.7 ± 1.5 mV in young rats (*n* = 6 and 8, respectively, *p* = 0.61, unpaired Student's *t*‐test). The mixture of blockers without XE991 depolarized the membrane potential of arterial smooth muscle cells (Figure 8B,D), wherein the level of this depolarization was comparable between adult and young arteries (Figure 8F). Addition of SNP led to a hyperpolarization, which was followed by depolarization after treatment with XE991 (Figure 8B,D). Importantly, arterial smooth muscle of young rats demonstrated larger changes of membrane potential in response to SNP as well as XE991 (Figure 8F).

## 4 DISCUSSION

This study provides novel data on the contribution of potassium channels to NO‐induced relaxation during postnatal development. We showed that the NO‐donor SNP had an anticontractile effect on rat saphenous arteries after functional elimination or inhibition, respectively, of available potassium channels which was similar in both adult and young rats. Thus, the anticontractile effect of SNP under these conditions was independent of potassium channels. A solely potassium channel‐independent anticontractile effect of SNP was observed also when either Kir6, or Kir2, or Kv2 channels, respectively, were available in both adult and young rats. However, when Kv1 channels were available, a Kv1 channel‐dependent component contributed to the anticontractile effect of SNP in young rats. Further, when BK_Ca_ channels were available, a BK_Ca_ channel‐dependent component contributed to the anticontractile effect of SNP in adult rats. Moreover, a considerable Kv7 channel‐dependent component contributed to the anticontractile effect of SNP in both adult and young rats.

### 4.1 Kv1, Kv2, Kv7, Kir2, Kir6, and BK_Ca_ channels are highly expressed in saphenous arteries of both adult and young rats

It has been shown that NO‐induced dilation can be mediated by different potassium channels like voltage‐gated potassium channels,^21^ in particular Kv7 channels^22^; BK_Ca_ channels^24^, ^25^; Kir2 channels,^26^ and Kir6 channels.^27^ The data of the present study show that at least some isoforms of Kv1 (*Kcna2, 5*), Kv2 (*Kcnb1*), Kv7 (*Kcnq1, 4, 5*), Kir2 (*Kcnj2*, 12), Kir6 (*Kcnj*8), and BK_Ca_ (*Kcnma1*) channels are expressed to a considerable extent at the mRNA level in saphenous arteries of both adult and young animals. Previously, we had shown differential mRNA expression of potassium channels in saphenous arteries of adult and young rats.^15^ Importantly, differential mRNA expression was also observed for some potassium channel regulatory subunits which have an activating influence on the pore‐forming subunits. In addition, despite lower Kv7.4 channel mRNA expression, higher Kv7.4 channel protein expression was found in young rats. The cited data show that a comprehensive insight into potassium channel expression and its possible relevance for the functional data shown in the present study also requires knowledge of channel expression at the protein level including regulatory subunits. However, the latter is beyond the scope of the present investigation and should be addressed in a future study. Nevertheless, the expression data obtained in the present study fully fulfill our intended purpose, namely to show that for the potassium channels known to potentially mediate SNP‐induced vasodilation, at least some isoforms are expressed in saphenous arteries of both adult and young rats.

### 4.2 The NO donor SNP relaxes rat saphenous arteries

The NO‐donor SNP was used in the present study. This classical nitrovasodilator has been shown to evoke vessel dilations closely resembling effects of NO released from the endothelium.^43^ Of note, NO‐independent effects of NO‐donors have been reported.^44^ However, previous studies established that the stimulatory effect of SNP on BK_Ca_ currents^45^ and the relaxing effect of SNP on rat tail arteries^26^, ^40^ were abolished in the presence of the NO‐scavenger hydroxocobalamin.^46^ In addition, neither cyanide, which could also be released by SNP,^39^ nor nitroxyl (HNO), which may be generated by the direct interaction of H_2_S with SNP^47^ contributed to the relaxing effect of SNP in rat tail arteries.^26^, ^40^ Thus, data obtained at the molecular, cellular, and organ level consistently suggest that the effect of SNP on vessel preparations is mediated by NO released from SNP.

### 4.3 Potassium channels contribute to the anticontractile effect of the NO donor SNP

In the present study, vessels were contracted with 50 mM KCl. This increases the extracellular concentration of potassium ions, so that the electrochemical gradient for potassium becomes greatly reduced. Under these conditions, vessel reactivity is largely independent of potassium channels, that is, potassium channels are functionally eliminated. This notion is supported by our observation that the depolarization accompanying the 50 mM KCl‐induced contraction was not affected during the SNP‐induced dilation. In addition, vessels were contracted by a mixture of potassium channel blockers (DPO‐1, stromatoxin, XE991, BaCl_2_, glibenclamide, and iberiotoxin). The latter contraction was similar to that seen in vessels contracted with 50 mM potassium chloride. This finding suggests that the blocker mixture most likely inhibited all functionally available potassium channels. Furthermore, SNP had an anticontractile effect on rat saphenous arteries after both functional elimination (pre‐contraction by KCl) or inhibition (pre‐contraction by blocker mixture) of available potassium channels. These findings suggest that the blocker mixture mimics the effect of functional removal of potassium channels by 50 mM KCl, at least with respect to the anticontractile effect of SNP.

Our data obtained after pre‐contraction by KCl or by the blocker mixture, respectively, also show that part of the anticontractile effect of SNP was independent of potassium channels. Indeed, NO has been shown to decrease the intracellular calcium concentration,^48^ in particular by reducing calcium influx via voltage‐gated calcium channels.^49^ In addition, NO reduces the calcium sensitivity of the contractile apparatus, (see e.g.,^50^, ^51^). However, these mechanisms and their possible maturational changes were not the focus of the presented investigation and have to be addressed in future studies.

### 4.4 Kir2, Kir6, and Kv2 channels do not contribute to the anticontractile effect of the NO donor SNP

Administration of a potassium channel blocker mixture *without* barium, glibenclamide, or stromatoxin contracted arterial segments in both adult and young rats. This blocker mixture inhibits dominant potassium conductances (e.g., BK_Ca_ and Kv7 channels), which we had shown can mask the functional availability of subordinate potassium conductances.^14^ The use of this blocker mixture should therefore have facilitated the functional occurrence of subordinate potassium conductances.

The central observation of the present study was that the SNP‐induced attenuation of the contraction produced by a potassium channel blocker mixture that leaves Kir2 or Kir6 or Kv2 channels available was similar in both adult and young rats compared with the effect of SNP on the contraction induced by 50 mM KCl. Since the contraction induced by 50 mM KCl provides conditions under which the effect of SNP is determined by potassium channel‐independent mechanisms, this suggests that Kir2, Kir6, and Kv2 channels do not contribute to the effect of SNP in the saphenous artery.

This observation is in contrast to previous reports, albeit from different vessels, showing that NO‐induced dilation can be mediated by Kir2,^26^ Kir6,^27^, ^52^ or Kv channels.^21^, ^53^ In agreement with the results of the present study, Kir6 channel‐resistant NO‐induced vasodilatation was also observed.^54^, ^55^ As mentioned previously,^18^ experimental conditions, methodology, the species and the vascular bed studied may determine the functional availability of these channels. In relation to saphenous arteries, functionally active Kir2 but not Kv2 channels were observed in vessels of both adult and young rats, albeit under different conditions than in the present study.^15^ In addition, previous research demonstrated Kir6 channel currents in freshly isolated smooth muscle cells from rat saphenous arteries.^56^

In conclusion, previous studies have shown that Kir2 and Kir6 channels in saphenous arteries can contribute to vessel tone regulation under certain conditions. The present study demonstrates that these channels were not required for the anticontractile effect of SNP even under conditions that facilitate the observation of subordinate potassium conductances. Kv2 channels were also not required for the anticontractile effect of SNP in saphenous arteries; their functional role in this vessel is generally unknown. Future studies are needed to clarify whether suitable conditions, if any, exist for the activation of Kir2, Kir6, and Kv2 channels by SNP in these vessels.

### 4.5 Kv7 and BK_Ca_ channels contribute to the anticontractile effect of the NO donor SNP in adult, but Kv7 and Kv1 channels contribute in young rats

The central observations of the present study were as follows: the SNP‐induced attenuation of the contraction produced by a potassium channel blocker mixture that leaves BK_Ca_ channels available was similar in young but stronger in adult rats compared with the effect of SNP on the contraction induced by 50 mM potassium chloride. This suggests that BK_Ca_ channels contribute to the effect of SNP in adult but not in young rats.

Further, the SNP‐induced attenuation of the contraction produced by a potassium channel blocker mixture that leaves Kv7 channels available was stronger in both adult and young rats compared with the effect of SNP on the contraction induced by 50 mM potassium chloride. Moreover, the effect of SNP on the contraction produced by a potassium channel blocker mixture that leaves Kv7 channels available was accompanied by membrane potential hyperpolarization in both adult and young rats, whereas the SNP‐induced attenuation of the contraction induced by 50 mM potassium chloride was not. Furthermore, the strong contractile and depolarizing effect of re‐addition of XE991 to the blocker mixture that leaves Kv7 channels available in the presence of SNP additionally suggests that Kv7 channels contribute to the SNP‐induced vasodilation in young and adult rats. Of note, the mRNA expression of Kv7.1 channels was considerably smaller than that of Kv7.4 channels. In functional experiments it was shown that Kv7.1 channels do not mediate ANP‐ and CNP‐induced dilation in rat aorta and renal artery^22^ and SNP‐ and GTN‐induced dilation in rat intrapulmonary arteries.^57^ Thus, Kv7.4/7.5 and not Kv7.1 channels most likely mediate the effect of NO.

Of note, a recent publication^58^ suggested that Kv7 channels are not required for cGMP‐mediated vasorelaxation, at least in mouse mesenteric arteries. However, as documented in detail in,^18^ experimental conditions, methodology, the species and the vascular bed studied may determine the functional availability of potassium channels. Indeed, NO‐induced dilation was shown to be mediated by Kv7 channels,^22^, ^59^ but not in all vessels or species studied.^22^, ^23^ Thus, the cited publication is not in direct contradiction to our findings obtained in rat saphenous arteries. In our study, the contribution of Kv7 channels was inferred from the effect of the Kv7 channel blocker XE991. The use of XE991 was based on reports in the literature but above all on our own intensive studies on the functional role of vascular smooth muscle potassium channels. Thus, the concentration employed, 3 μM, produced maximum effect on potassium channel opener induced vasodilation,^60^ showed differential effects in different vessels not consistent with a broadly unspecific effect^60^ and demonstrated interactions with several potassium channel openers and blockers inconsistent with nonspecific actions.^14^

Finally, the SNP‐induced attenuation of the contraction produced by a potassium channel blocker mixture that leaves Kv1 channels available was similar in adult rats but stronger in young rats compared with the effect of SNP on the contraction induced by 50 mM potassium chloride. Furthermore, in young rats, the strong contractile effect of DPO‐1 addition to the blocker mixture without DPO‐1 and stromatoxin in the presence of SNP suggests that Kv1 channels contribute to the SNP‐induced vasodilation in these rats. This suggests that Kv1 channels contribute to the effect of SNP in young but not in adult rats in the saphenous artery.

Interestingly, the contraction and depolarization induced by the blocker mixture that leaves Kv7 channels available, and the SNP‐induced relaxation of the contraction induced by this blocker mixture, as well as the contraction induced by re‐addition of XE991 to the blocker mixture in the presence of SNP, were similar in adult and young rats. In contrast, the SNP‐induced decrease of the depolarization induced by this blocker mixture, as well as the depolarization induced by re‐addition of XE991 to the blocker mixture in the presence of SNP, were larger in young than in adult rats. Thus, with respect to saphenous arteries exposed to SNP, these vessels of young rats require larger membrane potential changes for a similar change in vessel tone. The reason for this difference is currently not clear. However, this new observation, which was made for the first time in this study, lays the foundation for new hypotheses to be investigated in future studies.

The findings of the present study are consistent with previous reports demonstrating that NO‐induced dilation can be mediated by BK_Ca_ channels in many (e.g.,^24^, ^25^), but not all (e.g.,^61^, ^62^) vascular beds, by Kv7 channels^22^, ^59^ (but not in all vessels or species studied^22^, ^23^) as well as by Kv channels,^21^, ^53^, ^63^ although Kv channel subtypes were not addressed in these studies (for a comprehensive overview see e.g.,^18^). The data of the present study suggest that a differential contribution of BK_Ca_ and Kv1 channels to NO‐induced dilation can be explained by age‐related developmental processes, in addition to experimental conditions, methodology, the species and the vascular bed studied as mentioned above. Indeed, differences in the expression of BK_Ca_ channel subunits have been shown by us in previous studies on saphenous arteries, with BK_Ca_ channels expressed at lower levels in young than in adult rats.^14^, ^15^ In addition, due to their dominance in young rats, Kv7 channels may cause a stronger suppression of the functional impact of BK_Ca_ channels in young compared to adult rats.^14^ Other possible mechanisms (e.g., developmental differences in the cGMP pathway) have to be explored in the future. In relation to saphenous arteries, functionally active Kv7, BK_Ca_, and Kv1 channels were observed in both adult and young rats, albeit under different conditions than in the present study where the activity of BK_Ca_ channels is large in adult but rather small in young rats and where the activity of Kv1 channels was observed only in young rats.^14^, ^15^

In conclusion, previous studies have shown that Kv7 and BK_Ca_ channels in saphenous arteries of both adult and young rats and Kv1 channels in young rats can contribute to vessel tone regulation under certain conditions. In adult rats, the functional role of Kv1 channels in saphenous arteries is currently unknown. The present study demonstrates that Kv7 and BK_Ca_ channels contribute to the anticontractile effect of SNP in adult, but Kv7 and Kv1 channels contribute in young rats. Future studies are needed to clarify whether suitable conditions, if any, exist for the activation of BK_Ca_ channels in young and of Kv1 channels in adult rats by SNP in these vessels.

### 4.6 Limitations of the present study

This study did not address the question how the NO‐donor SNP affects potassium channels, in particular Kv7 channels, during vasorelaxation. NO‐induced vasorelaxation depends mostly on soluble gyanylyl cyclase,^64^ leading to an increase of intracellular cGMP and stimulation of PKG, with the latter affecting a number of targets,^65^ including potassium channels (see^18^). SNP‐induced, Kv7 channel dependent relaxation has been reported previously,^22^, ^57^, ^59^, ^66^ and an involvement of the GC/PKG pathway was suggested using a selective PKG inhibitor,^22^ and a guanylyl cyclase inhinitor.^59^ The pathway involved and possible differences in adult and young rats was not the focus of the present study and should be addressed in the future.

The data of the present study, showing a shift in the potassium channels involved in NO‐induced vasorelaxation during postnatal development from Kv1 and Kv7 channels to BK_Ca_ and Kv7 channels, have been obtained from a single vessel, the saphenous artery of rats. It is therefore not clear whether such a shift also occurs in other vessels. Furthermore, the known vessel‐ and species‐specific differences in the involvement of potassium channels in NO‐induced vasorelaxation^18^ must be taken into account. If a shift in the involvement of potassium channels in NO‐induced vasorelaxation should also occur in other vessels, it is also unclear whether the pattern of the shift in participating potassium channels in other vessels or species is exactly the same or not. These questions arising from the new results of the present study need to be clarified in follow‐up studies.

## 5 CONCLUSION

**FIGURE 9:**
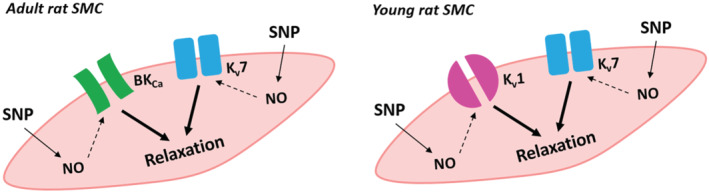
Scheme illustrating the key findings: Potassium channels, even multiple ones, contribute to SNP‐induced vasorelaxation in young and adult rats and these channels change from Kv1 and Kv7 channels to BK_Ca_ and Kv7 channels during postnatal development.

Saphenous arteries of both adult and young rats express at least some isoforms of potassium channel families that are known to potentially mediate SNP‐induced vasodilation. SNP induces an anticontractile effect in both adult and young rats. Kir2, Kir6, and Kv2 channels in both adult and young rats, BK_Ca_ channels in young rats, and Kv1 channels in adult rats do not contribute to this effect. In contrast, Kv7 channels in both adult and young rats, BK_Ca_ channels in adult rats, and Kv1 channels in young rats contribute to the anticontractile effect of SNP. Thus, the data of the present study show for the first time that potassium channels, even multiple ones, contribute to SNP‐induced vasorelaxation in newborn rats and that the potassium channels involved in SNP‐induced vasorelaxation change from Kv1 and Kv7 channels to BK_Ca_ and Kv7 channels during postnatal development (Figure 9).

## AUTHOR CONTRIBUTIONS

Anastasia A. Shvetsova, Dina K. Gaynullina, and Rudolf Schubert conceived and designed the research; Anastasia A. Shvetsova, Dina K. Gaynullina, Peter Winkler, and Paulus Wohlfart, performed the research and acquired the data; Anastasia A. Shvetsova, Dina K. Gaynullina, Peter Winkler, Paulus Wohlfart, and Rudolf Schubert analyzed and interpreted the data; All authors were involved in drafting and revising the manuscript.

## CONFLICT OF INTEREST STATEMENT

The authors declare no conflicts of interest.

## Data Availability

The data that support the findings of this study are available from the corresponding author upon reasonable request.
